# New sight at the organization of layers of multilayer polyelectrolyte microcapsules

**DOI:** 10.1038/s41598-021-93565-2

**Published:** 2021-07-07

**Authors:** Egor V. Musin, Aleksandr L. Kim, Alexey V. Dubrovskii, Sergey A. Tikhonenko

**Affiliations:** Institute of Theoretical and Experimental Biophysics, Russian Academy of Science, Institutskaya St., 3, Puschino, 142290 Moscow Region, Russia

**Keywords:** Biophysics, Materials science, Nanoscience and technology

## Abstract

In this work, the mutual arrangement of polyelectrolytes of multilayer polyelectrolyte microcapsules (with layers—[PAH/PSS]_3_PAH) by determination of the dissociation level of polyallylamine (PAH) from the surface of a polyelectrolyte microcapsules (PMC) of various types was studied: PMC with a dissolved CaCO_3_ core after preparation, PMC with an undissolved CaCO_3_ core and PMC with an encapsulated protein. It was concluded that the polyelectrolyte layers are mixed in the entire shell of the capsules with a dissolved CaCO_3_ core. In the case of the PMC with an undissolved CaCO_3_ core, such mixing of polyelectrolyte layers does not occur. That fact allows us to conclude that the mixing of polyelectrolytes layers mixing at the stage of dissolution of CaCO_3_ core. The PMC with encapsulated protein has partial mixing of polyelectrolytes layers. That phenomenon may be due to the fact that seven-layered protein-containing microcapsules already have a dense and well-formed shell. The obtained data correlate with the data on the study of the surface charge of microcapsules.

## Introduction

For the first time, polyelectrolyte microcapsules (PMC) were obtained in 1998, since then it is one of the most studying object of polymer nanotechnology^1,2^. Microcapsules have been created on LbL technology^3^, by alternately layering of positive and negative charged polyelectrolytes on dispersed particles with nano and micro size^1,4–6^. The polyelectrolyte shells of microcapsules are permeable for low molecular substances and ions, but they are not permeable for high molecular substances (more than 1 kDa)^7^. A distinctive feature of polyelectrolyte microcapsules is the possibility of including different substances: inorganic nanoparticles^8–12^, carbon nanotubes^13^, antibodies^14,15^, dyes^16–19^, quantum dots^20–22^ and etc.

The above-described properties of PMCs make it possible to use them as targeted drug delivery systems^7,23,24^ as well as to create a treatment with prolonged action with controlled release^10,25–27^. The possibility of creating theranostics based on PMC containing quantum dots for the fight against cancer is being active studied^22^. There are studies of the diagnostic system creating based on the polyelectrolyte microcapsules for determination of pH medium, the concentration of low molecular weight compounds^28–32^, as well as original methods for separating mixtures of various organic and inorganic substances, in particular, the separation of heavy metal ions from the medium and the determination of the surface charge^33–37^.

**Figure 1:**
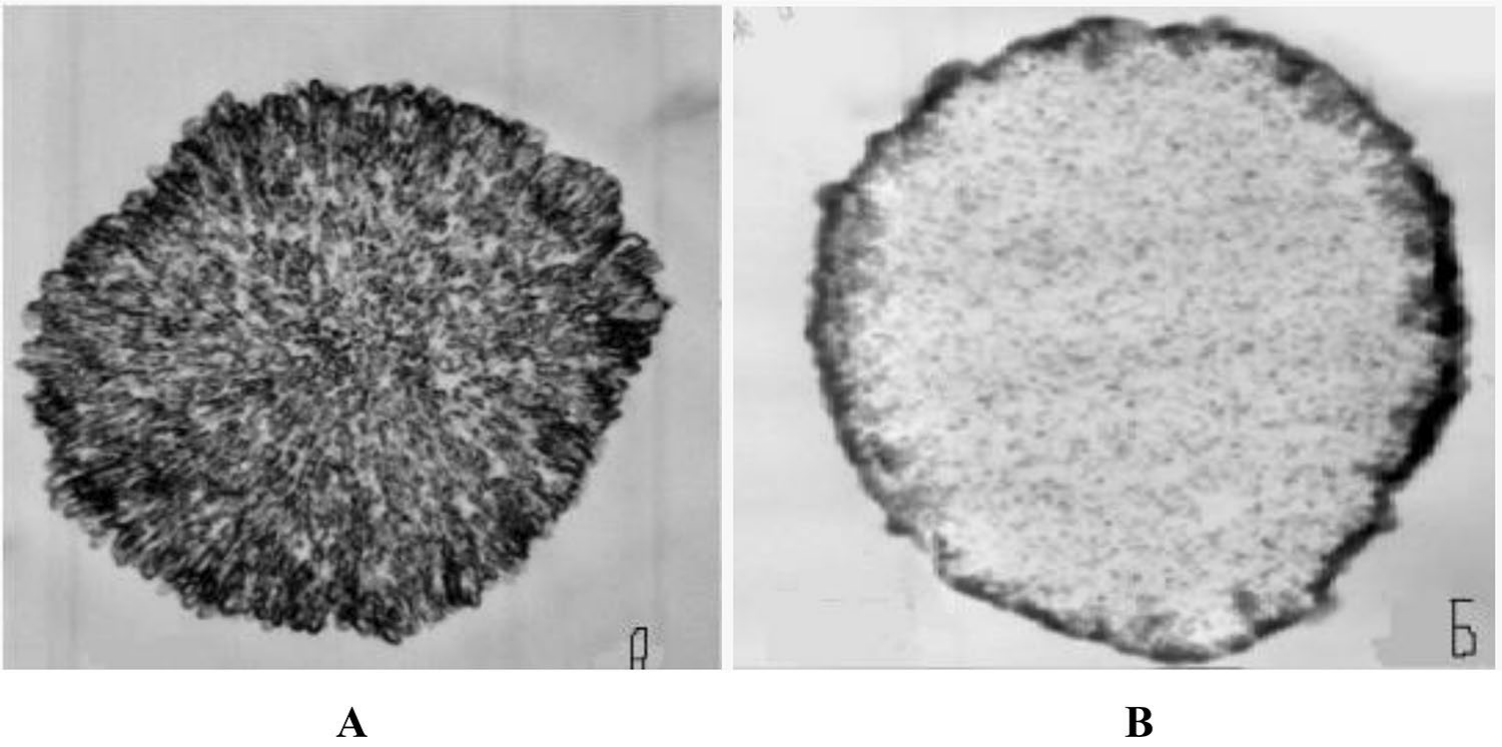
Electron microscopy of ultrathin slice of protein-free PMC (**A**) and PMC (**B**) containing protein^40^.

The polyelectrolyte microcapsules may be used in different fields: medicine, food and pharm industry. However, features of the internal structure of PMC^38,39^ and the mutual arrangement of polyelectrolytes in them are still unclear, despite studies devoted to PMC’s stability and its ultrastructural organization^40^. In the work of Kazakova et al. was shown that PMC with dissolved CaCO_3_ core and PMC with encapsulated protein have different inner structure^40^. The PMC with dissolved CaCO_3_ core is filled by interpolyelectrolyte complex (Fig. 1A) against the PMC containing protein (Fig. 1B) with a dense, well-formed shell.

Such research is required for understanding of mechanisms of interaction between encapsulated subject and polyelectrolytes, which part of polyelectrolyte shell. Also, it is necessary to predict PMC’s interaction in the medium, for example, the adhesion of microcapsules with surface, it’s immunogenetic and etc.

In this way, the aim of this work is to study the mutual arrangement of polyelectrolytes of PMC by the research of dissociation of shell layers of different types of PMC, consisting of polyallilamyne (PAH) and polystyrene sulfonate (PSS).

## Materials and methods

Polystyrenesulfonate sodium (PSS) and polyallylamine hydrochloride (PAH) with a molecular mass of 70 kDa Sigma (Merck KGaA, Darmstadt, Germany), bovine serum albumin (BSA), fluorescein isothiocyanate (FITC) Sigma (Merck KGaA, Darmstadt, Germany); ethylenediaminetetraacetic acid (EDTA), calcium chloride (CaCl_2_ × 2H_2_O), sodium chloride and sodium carbonate from Reahim (Reahim AO, St.Petersburg, Russian Federation) were used.

### Preparation of fluorescently labeled PAH

FITC was slowly added to a stirring (300–400 rpm) solution of polyelectrolyte (10 mg/mL) in 50 mM borate buffer, pH 9.0. The components were fused in a molar ratio of FITC: PAH (BSA) (proportion of FITC to amino groups of BSA) = 1: 100. After that, its solution was incubated for 1.5–2 h. After incubation, the solution was dialyzed against water (10 L) overnight.

### Preparation of CaCO_3_ microspherolites

0.33 M Na_2_CO_3_ solution was rapidly added to the 0.33 M CaCl_2_ stirring solution (which contained 3 mg/mL of BSA if it was necessary). The stirring was continued for 30 s. The suspension was maintained until complete precipitation of the formed particles. The process of "ripening" of microspherolites was controlled with the help of a light microscope. Then, the supernatant was decanted, the precipitate was washed with water and used to prepare PMC. The microparticles were obtained with an average diameter of 5 μm.

### Preparation of polyelectrolyte microcapsules

**Figure 2:**
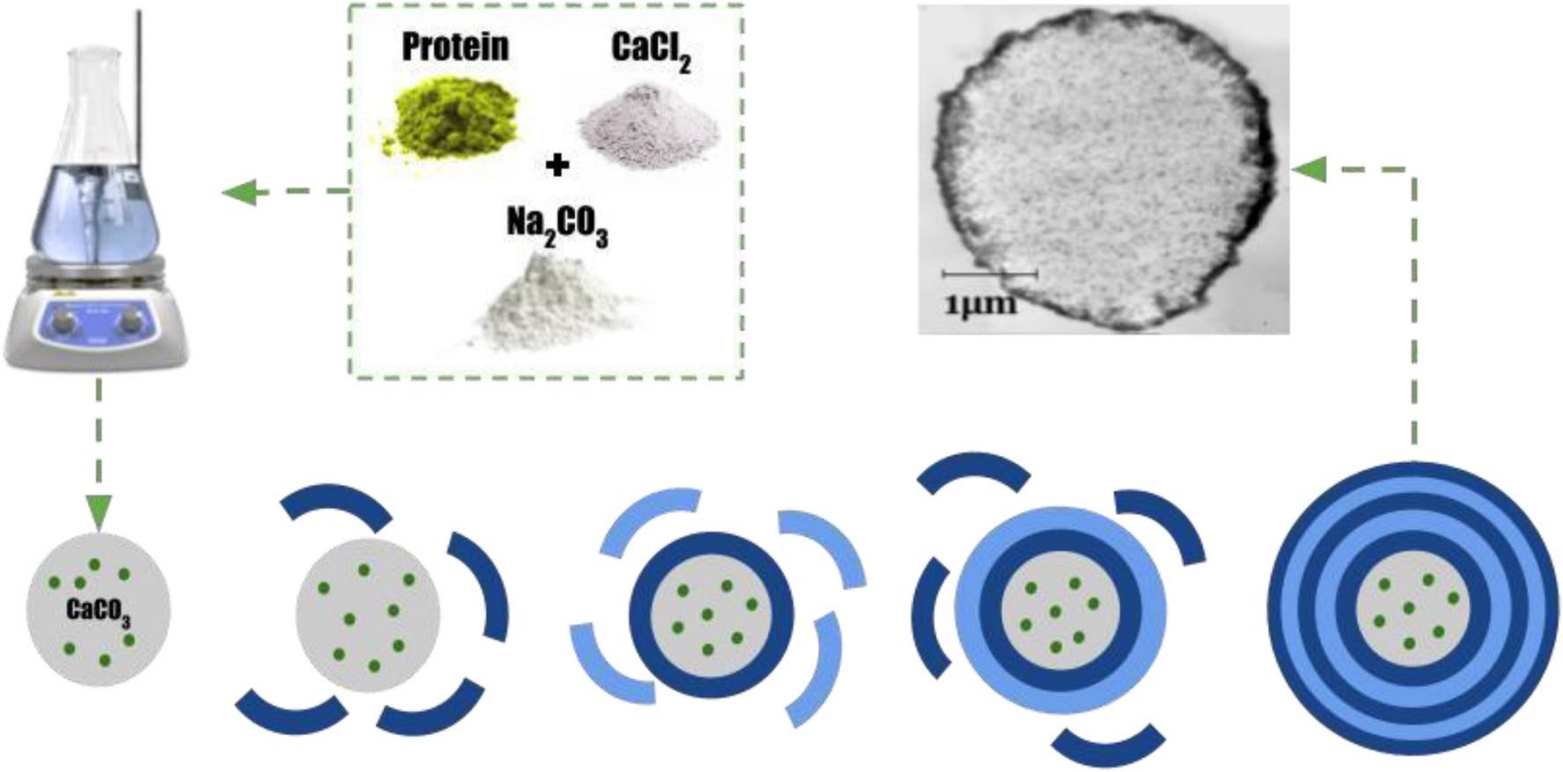
Stages of polyelectrolyte microcapsules preparation.

The “sponge” type of polyelectrolyte microcapsules were obtained by alternately adsorbing the oppositely charged polyelectrolytes onto a dispersed microparticle (core), followed by dissolution of this cores. At the moment of dissolution of CaCO_3_ core the inner space of PMC is filled by interpolyelectrolyte complex^40^. The microcapsule production process is shown in Fig. 2. Alternate adsorption of PSS and PAH on the surface of CaCO_3_ microspherolites was carried out in solutions of polyelectrolytes with a concentration of 2 mg/mL containing 0.5 M NaCl. Each step of adsorption was followed by a triple wash with a 0.5 M NaCl solution, which was necessary to remove unabsorbed polymer molecules. The particles were separated from the supernatant by centrifugation. After applying the required number of layers, the carbonate kernels were dissolved in a 0.2 M EDTA solution for 12 h. The resulting capsules were washed three times with water to remove core decay products. Depending on the experiment capsules containing labeled PAH as 1, 3, 5, 7th layers were obtained. In the case of the PMC with an encapsulated protein, we used a protein filled CaCO3 core which is dissolved at the final stage of preparation. In the case of the PMC with an undissolved CaCO_3_ core, the last stage of preparation is absent.

### Registration of an envelope dissociation of FITC-labeled PAH from polyelectrolyte capsules

A microcapsules envelope dissociation were analyzed by fluorescent spectroscopy. The polyelectrolyte microcapsules containing FITC-labeled PAH in their envelopes and encapsulating FITC-labeled BSA were centrifuged at 3,000 rpm for one minute. Fluorescence of the supernatant was measured. The fluorescence spectra were registered with Cary Eclipse (USA) in a thermal controlled cuvette with 1 cm path length at light excitation with 273 nm wavelength.

### Determination of the PMC charge

The charge of microcapsules was determined with a Zetasizer nano ZS device (Malvern, UK) and a set of standard plastic disposable cuvettes for sizing (DTS0012) and cuvettes for measuring zeta potential and conductivity (DTS1060 or DTS1070). The measurements were carried out in distilled water at + 25C. The scattering angle of the laser light is recorded at 173°. The accumulation time of the correlation function is 15 cycles of 15 s. The number of measurement repetitions is at least 3. The Attenuator is set in the 8–11 range. Henry's function value is 1.5. The potential difference applied to the cuvette electrodes is 70 mV.

## Results and discussion

At the first step of our research, we studied the surface charge of three types of polyelectrolyte microcapsules: PMC contained a protein, PMC with dissolved CaCO_3_ core filled with an interpolyelectrolyte complex and PMC with an undissolved CaCO_3_ core. Also, polyelectrolyte microcapsules were prepared with different outer layers: a PSS-polyanion and a PAH-polycation. The results of this study are shown in Table 1.

**Table 1:** Results of the study of the surface charge of polyelectrolyte microcapsules.

	Microcapsule type		
	PMC with CaCO_3_ core	PMC with interpolyelectrolyte complex	PMC with protein
[PAH/PSS]_3_	− 20 ± 3.4 mV	+ 25 ± 3.1 mV	+ 22 ± 1.9 mV
[PAH/PSS]_3_PAH	+ 24 ± 2.7 mV	+ 23 ± 3.4 mV	+ 20 ± 3.6 mV

It can be seen from the table that the surface charge of the PMC does not depend on the charge of the polymer forming the outer layer of the capsule in all cases, except for microcapsules with a not dissolved CaCO_3_ core. In this regard, we put forward a hypothesis that the different mutual arrangements of polyelectrolytes of the different types of the microcapsule.

We carried out several studies on the dissociation of PMCs containing FITC-labeled PAH to test this hypothesis. As an object of research, we used three types of seven-layer polyelectrolyte microcapsules: filled with protein, filled with an interpolyelectrolyte complex, and capsules containing a CaCO_3_ core. Polyelectrolyte microcapsules contained FITC-labeled PAH in one of the following layers: layers 1, 3, 5, 7.

Proceeding from the fact that the polyelectrolyte dissociates only from the surface of the PMC, in this way, we can assume that if the polyelectrolyte layers are not mixing, we will notice the dissociation of only the upper layer of the capsule. On the other side, if we observe the dissociation of another layer, including the lower layers, then this will indicate that polyelectrolyte layers were mixed.

We have previously shown that the dissociation of PMC depends on the ionic strength of the solution, due to the electrostatic nature of the interpolyelectrolyte interactions^38,39^. Thus, polyelectrolyte microcapsules incubated in a 2 M NaCl solution will be observed a more pronounced release of polyelectrolyte into solution.

**Figure 3:**
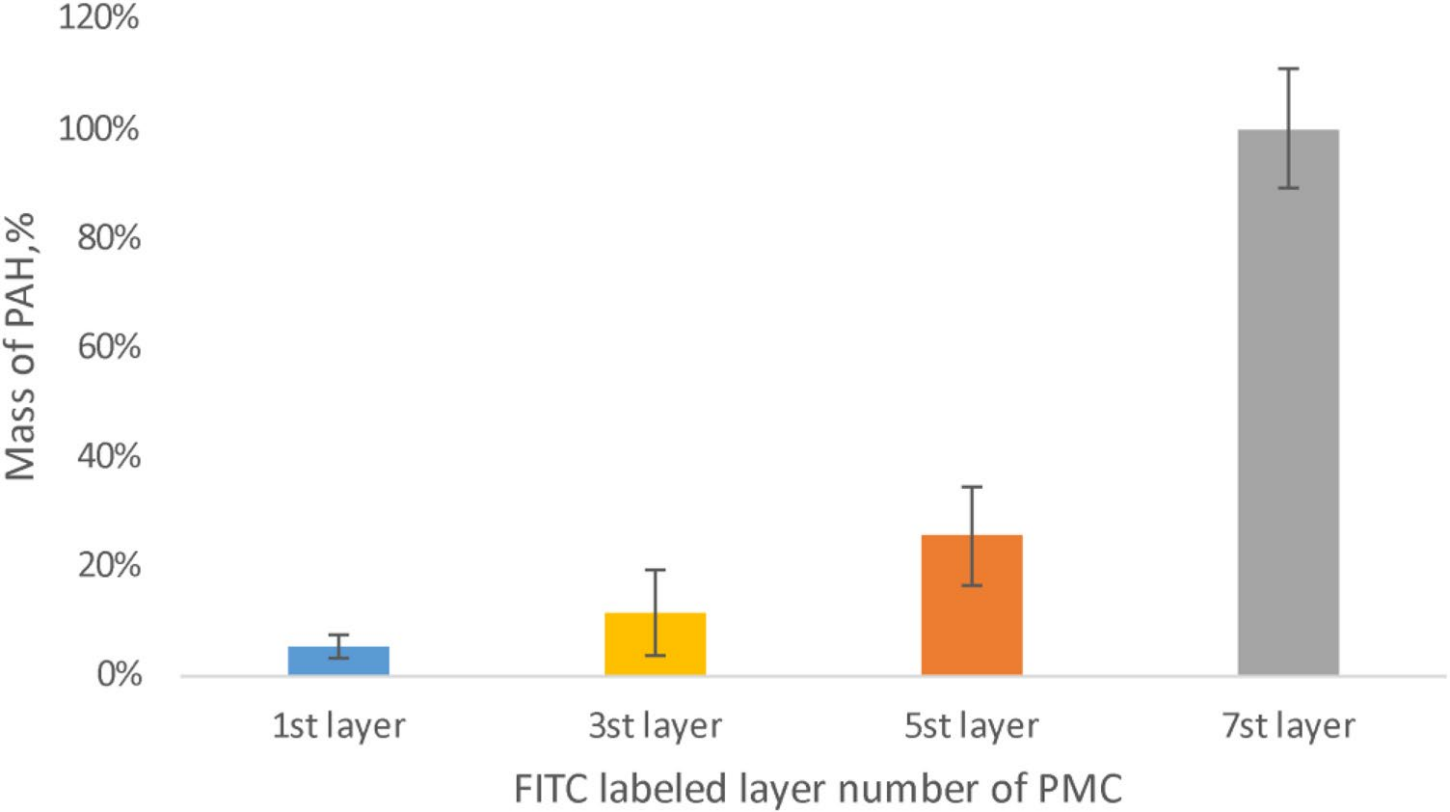
Dissociation of polyelectrolyte layers of microcapsules contained CaCO3, incubated in 2 M NaCl solution.

Studying of polyelectrolyte microcapsules contained CaCO3. The results of the study of capsules containing CaCO3 are shown in Fig. 3.

Hereinafter, the amount of polymer released into the solution of polyelectrolyte microcapsules with the labeled 7th layer was taken as a value of 100%. This is necessary to compare the dissociation of labeled PAH from different layers with each other. In the case of the microcapsules that contained the CaCO_3_ core (Fig. 3), it has been shown that the most pronounced dissociation of the outer layer (7th). Also, that has been shown that the first and the third labeled PAH layers of PMC less released into the solution. So, it can be concluded that the lower layers of polyelectrolytes are partly fixed by calcium carbonate core inside a polyelectrolyte microcapsule, thus the mobility of polyelectrolytes depends on the depth of occurrence and weakens from the inner layer to the outer layer.

### Studying of polyelectrolyte microcapsules filled with an interpolyelectrolyte complex

**Figure 4:**
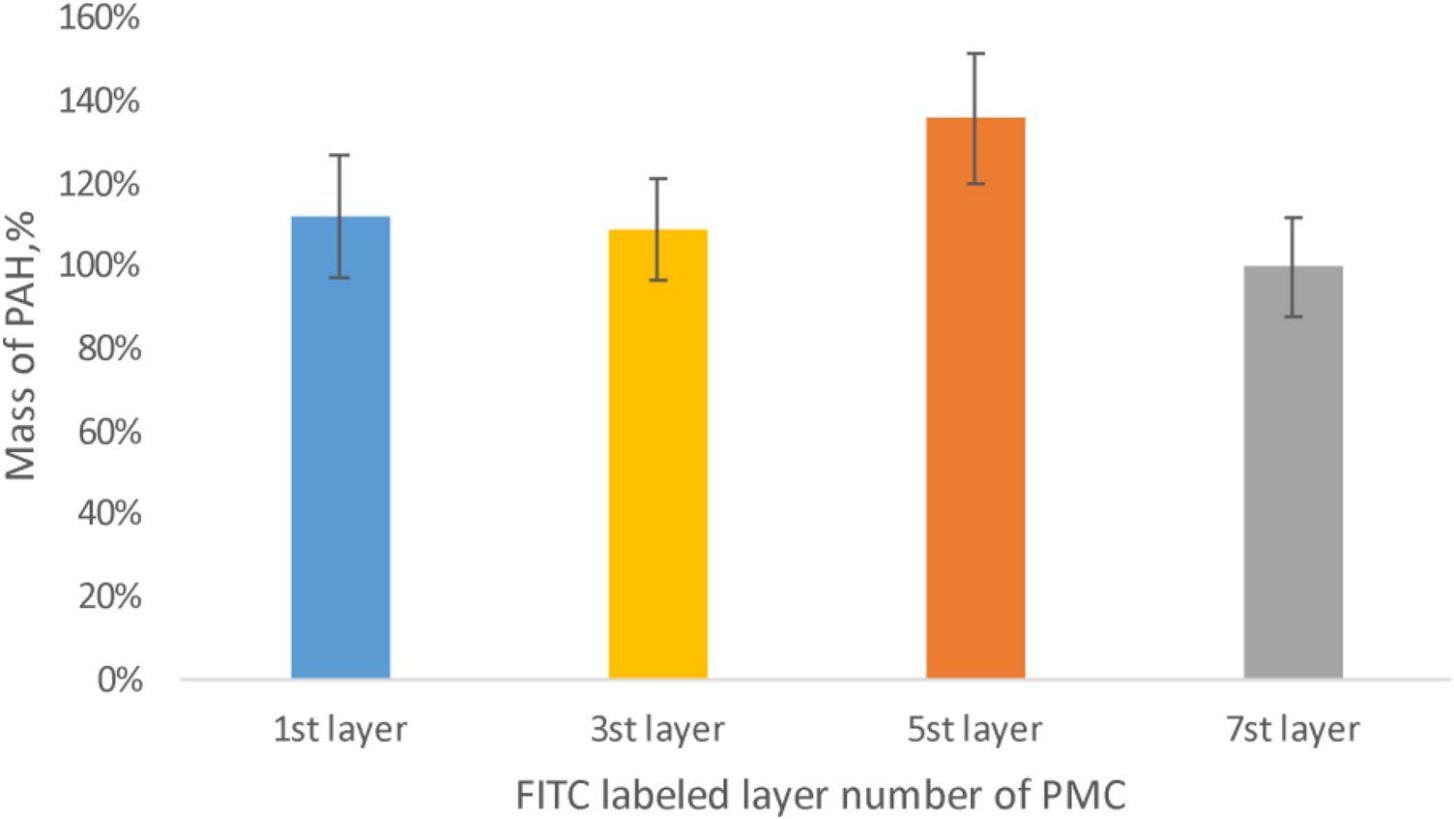
Dissociation of polyelectrolyte layers of microcapsules, which contained interpolyelectrolyte complex, incubated in 2 M NaCl solution.

In the case of capsules filled with an interpolyelectrolyte complex (Fig. 4), we observe an approximately equal amount of the released polymer of each labeled layer. Thus, we compared the obtained result of sponge capsules with dissolved the CaCO_3_ core, which has an inner spongy structure^41^ and defines the structure of PMC before dissolving^42^. We can conclude that the removal of the CaCO_3_ core leads to disruption of the capsule structure and mixing of the polyelectrolyte layers with each other.

### Studying of polyelectrolyte microcapsules filled with protein

**Figure 5:**
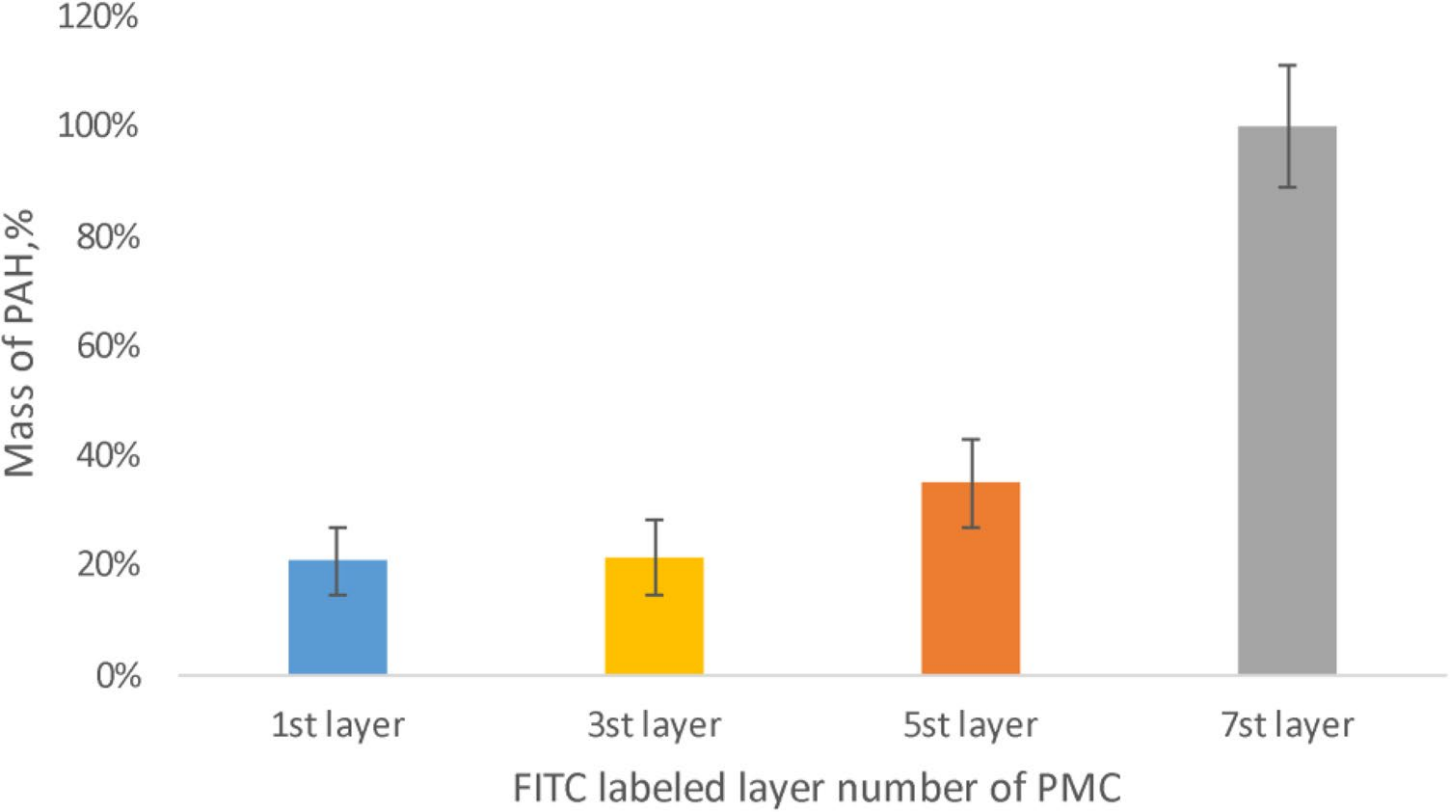
Dissociation of polyelectrolyte layers of microcapsules contained protein, incubated in 2 M NaCl solution.

Subsequently, a study of polyelectrolyte microcapsules contained protein was conducted results have shown in Fig. 5.

The figure shows that the most pronounced dissociation of PAH occurs from the 7th layer of the microcapsule. However, in the case of 1, 3, and 5 labeled layers, a slight dissociation of the polyelectrolyte is also observed. Considering the change in the surface charge of such capsules, demonstrated above, it can be concluded that in the case of dissolution of the core containing the protein, partial mixing of the polyelectrolyte layers occurs. This may be due to the fact that protein-containing microcapsules already at 7 layers have a dense, well-formed shell, in contrast to spongy microcapsules^40^, which prevents complete mixing of polyelectrolytes.

## Conclusion

In this work, the mutual arrangement of polyelectrolytes of PMC by the research of dissociation of shell layers of different types of PMC was studied: PMC with dissolved CaCO3 contained an interpolyelectrolyte complex, PMC contained a CaCO3 core and PMC contained a protein. For this study, the polyelectrolyte microcapsules contain FITC-labeled layers of PAH (layers: 1, 3, 5, 7) were made.

It was found that the dissociation of PAH in microcapsules with dissolved CaCO3 core contained the interpolyelectrolyte complex is approximately the same and does not depend on the depth of the labelled polyelectrolyte. It can be concluded that the PMC has the polyelectrolyte layers are mixed in the entire cavity of the capsules. Dissociation of PAH in microcapsules containing protein and CaCO3, was observed mainly in the 7th (upper) layer. The PMC contained CaCO3 does not occur such mixing, which indicates the mixing of polyelectrolytes at the stage of dissolvation of CaCO3 core. In the PMC contained protein, in the case of 1, 3, and 5 labeled layers, a slight dissociation of the polyelectrolyte is also observed. For that reason, it was concluded that partial mixing of polyelectrolytes occurs, which may be due to the fact that protein-containing microcapsules already at 7 layers have a dense and well-formed shell, which prevents complete mixing of polyelectrolytes.

Also, It has been studied that the surface charge of the PMC does not depend on the charge of the polymer forming the outer layer of the capsule. That fact observed in each case, except for PMC with an undissolved CaCO3 core. The data obtained correlate with the data on the study of the surface charge of microcapsules.
